# Endometrioid adenocarcinoma arising in adenomyosis in a patient with pelvic organ prolapse—case report

**DOI:** 10.1186/s12905-023-02310-6

**Published:** 2023-03-30

**Authors:** Jing Wang, Qingyuan Wang, Wenyan Wang, Jian Yang, Jingxian Xia, Yanan Wei

**Affiliations:** 1 Department of Obstetrics and Gynecology, The Second Affiliated Hospital of Anhui Medical University, Hefei, Anhui China; 2 Department of Obstetrics and Gynecology, Hefei Cancer Hospital, Chinese Academy of Sciences, Hefei, Anhui China

**Keywords:** Adenomyosis, Endometrioid adenocarcinoma, Endometriosis, Case report

## Abstract

**Background:**

Adenomyosis is a frequent finding in endometrial carcinoma patients. Endometrioid adenocarcinoma is the most common type of endometrial carcinoma; however, endometrioid adenocarcinoma arising from adenomyosis is extremely rare.

**Case presentation:**

In this case report, we describe a 69-year-old woman who required surgical treatment for pelvic organ prolapse (POP). The patient had been postmenopausal for 20 years and had no abnormal bleeding after menopause. The patient underwent transvaginal hysterectomy, repair of anterior and posterior vaginal walls, ischium fascial fixation and repair of an old perineal laceration. Histological examination of surgical specimens revealed endometrioid adenocarcinoma of the uterus. Bilateral adnexectomy, pelvic lymphadenectomy and para-aortic lymphadenectomy were then performed. The postoperative histopathological diagnosis was stage IB endometrial cancer (endometrioid carcinoma G2).

**Conclusions:**

In summary, endometrioid adenocarcinoma arising from adenomyosis (EC-AIA) is a rare entity and the early diagnosis is difficult. Adequate preoperative assessment and enhanced inquiry of occult clinical symptoms of postmenopausal women before hysterectomy may contribute to the diagnosis of EC-AIA preoperatively.

## Background

Endometrial carcinoma (EC) occurs in approximately 2.8% of women [1], and mortality rates have been increasing by 1.9% per year on average [2]. Endometrioid adenocarcinoma is the most common type of EC [3]. Risk factors for EC include body mass index (BMI), hypertension, hyperinsulinaemia and prolonged exposure to unopposed oestrogen [2]. Surgery remains the mainstay of the management for endometrial carcinoma [4]. Adenomyosis is a common benign histopathology found in the hysterectomy specimens of patients with endometrial carcinoma, and the incidence of endometrial carcinoma coexisting with adenomyosis (EC-A) varies from 0 to 70% [4]. However, EC arising in adenomyosis (EC-AIA), i.e., malignant transformation of adenomyosis to adenocarcinoma, is extremely rare and the incidence of EC-AIA is reported to be 1.35% [5]. When EC-AIA occurs only in the myometrium without endometrial involvement, it is easy to distinguish EC-AIA form EC-A according to the pathological diagnostic criteria established by Colman and Rosenthal [6]. However, when the endometrium is involved, EC-AIA no longer meets the above strict diagnostic criteria and it is difficult to differentiate these two different entities. Although adenomyosis was proven to have a protective effect against cancer progression in a recent study, EC-AIA is thought to be associated with poor survival [1]. Due to the low incidence, the current research is limited to a series of case reports. The clinical features and prognosis of EC-AIA are still unknown and the treatment of EC-AIA lacks authoritative guidelines [7].

Here, we report a case of endometrioid adenocarcinoma arising in adenomyosis incidentally diagnosed at the time of pelvic floor surgery in a 69-year-old female with no endometrial carcinoma risk factors. The patient provided written informed consent for the publication of information about herself without her name attached.

## Case presentation

A 69-year-old woman, gravida five and para four, presented with genital prolapse and no other clinical symptoms. The patient had been menopausal for 20 years, and she had no abnormal bleeding and no hormone replacement therapy after menopause. Her past menstrual history was normal and she did not have any other history of PCOS or oligomenorrhea or dysmenorrhea. The patient had no history of malignancy or family history of malignancies. She was admitted for operative treatment of pelvic organ prolapse (POP). Pelvic examinations using pelvic organ prolapse quantitation (POP-Q) revealed subtotal uterine prolapse (POP-Q III) along with anterior vaginal prolapse (POP-Q III), posterior vaginal prolapse (POP-Q I).

Preoperative transvaginal ultrasonography (TVS) showed that the endometrium was linear and that the size and shape of the uterus were appropriate for the patient’s age. There was no abnormal echo in the uterine cavity. The cervical smear and routine laboratory findings were normal. Tumor markers were not examined. As a benign lesion had been suspected preoperatively, neither CT nor MRI was arranged.

Three days after admission, the patient underwent transvaginal hysterectomy, anterior and posterior vaginal wall repair and ischium fascia fixation. Postoperative specimens showed that the mucosa of the uterine cavity and cervical canal was smooth.

**Fig. 1:**
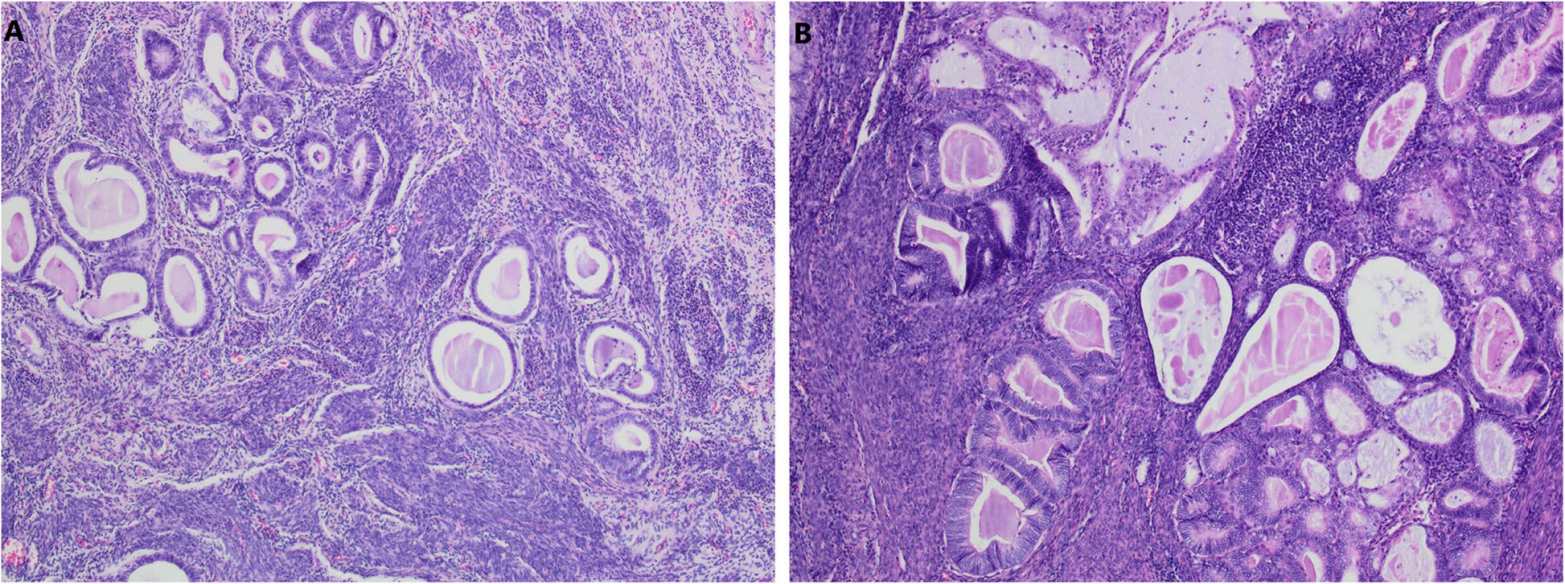
Endometrioid adenocarcinoma arising in adenomyosis. **A** Normal endometrial glands in adenomyosis (HE × 10); **B** Endometrioid adenocarcinoma and atypical lesions (HE × 10)

**Fig. 2:**
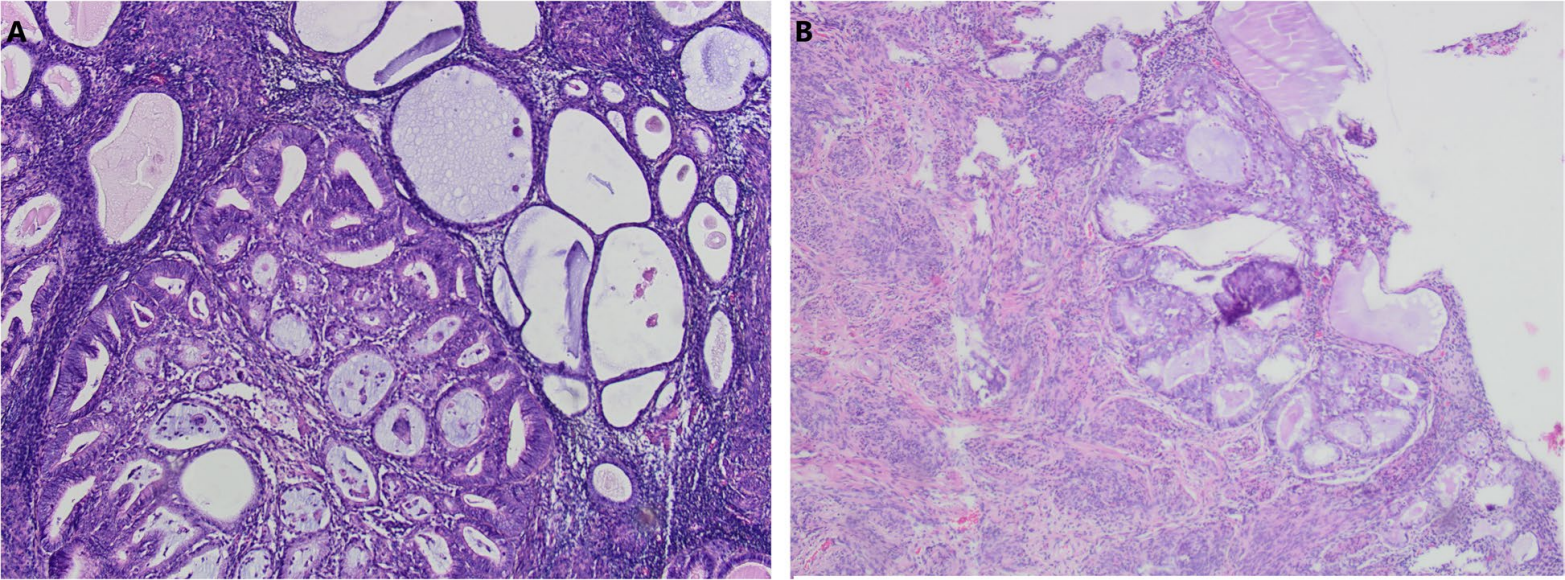
Histopathological appearances of endometrioid adenocarcinoma (HE × 10). **A** The malignant lesion in adenomyosis; **B** The malignant lesion in endometrium

**Fig. 3:**
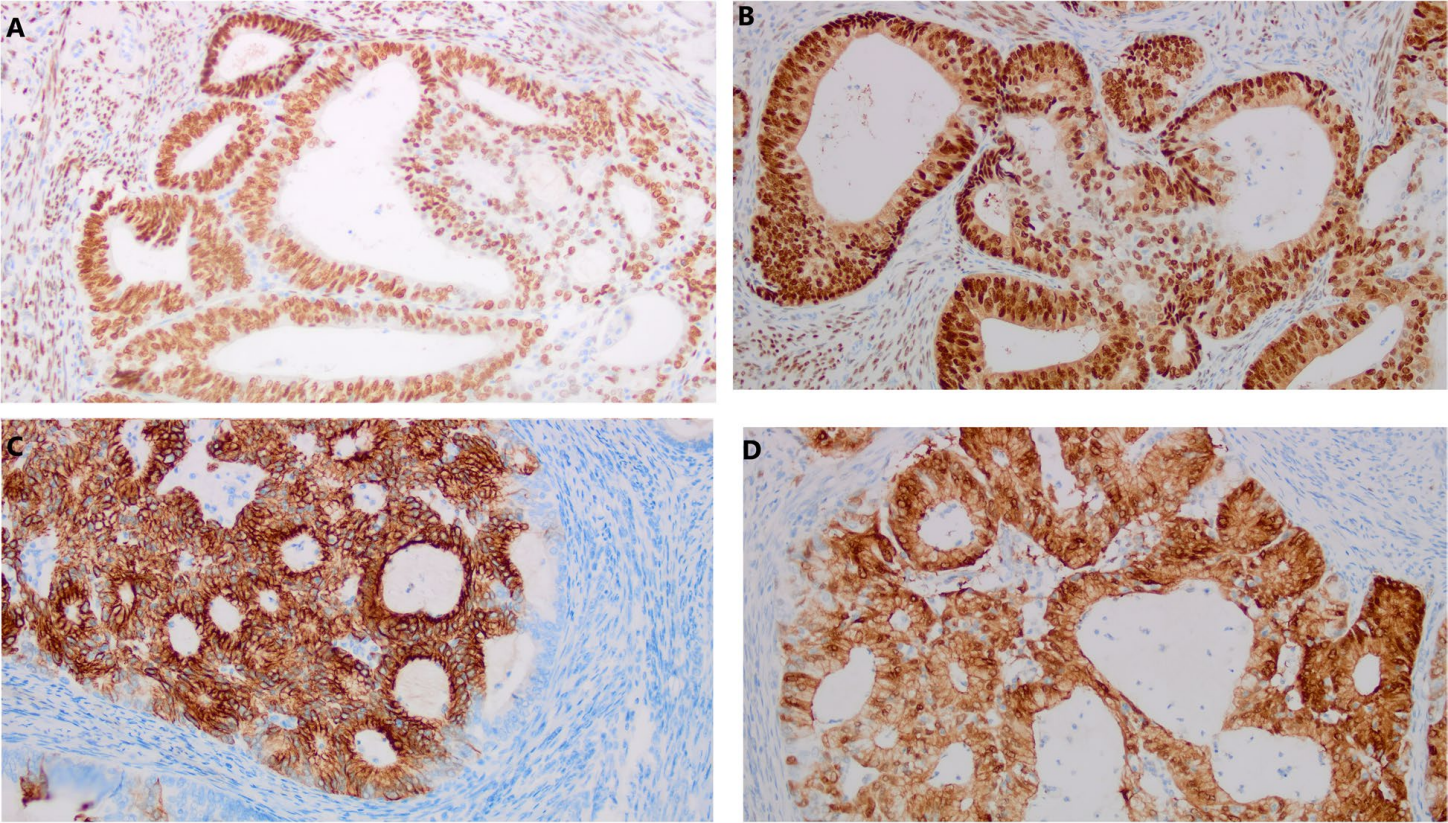
**A** Immunostaining with oestrogen (ERx200); **B** immunostaining with progesterone (PRx200); (**C**) immunostaining with CK-7 (× 200); (**D**) immunostaining with P16 (× 200)

**Fig. 4:**
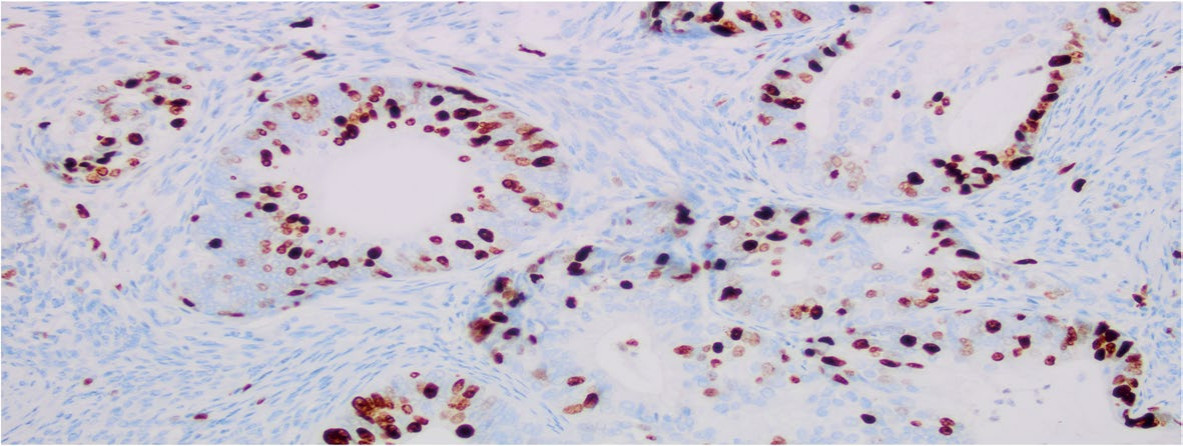
Immunostaining with Ki67 (× 200)

The postoperative gross specimen showed that the endometrium was thin. There was a tumorous lesion measuring 0.7 cm in diameter in the myometrium. Another one, measuring 1.0 cm in diameter, was found in the submucosa. The histopathology of the specimen revealed grade 2 endometrioid adenocarcinoma arising in adenomyosis (Fig. 1). The normally situated endometrium had a malignant lesion and the morphology of the carcinoma cells was the same as that of the carcinoma cells in adenomyosis (Fig. 2). Immunohistochemical results suggested that the carcinoma cells were positive for oestrogen receptor (ER), progesterone receptor (PR), CK7, P53, P16, CA125 and Pax-8 (Fig. 3). The mitotic index by Ki-67 immunostaining was approximately 40% (Fig. 4). Some carcinoma cells were positively stained for CEA and Vimentin, while a small number of them were positive for CK5/6. The depth of the tumour involvement was approximately 0.9 cm, and the thickness of the myometrium involvement was approximately 1/2 of the myometrium. No lymphovascular space invasion was found. Cervical and vaginal margins were not involved. The FIGO stage was TIBNxMx.

The patient was stable and was discharged 4 days after the operation. A month later, the patient was readmitted for surgery for endometrial carcinoma. Her preoperative serum CA125 and HE4 level were 10.4 U/mL and 66.4 U/mL, respectively. She underwent laparoscopic bilateral adnexectomy, pelvic lymphadenectomy and para-aortic lymphadenectomy. During the operation, the pelvic and abdominal cavity was explored without any obvious abnormal findings. Postoperative pathology showed that the bilateral adnexa, pelvic lymph nodes and para-aortic lymph nodes were not involved. No malignant cells were found in the peritoneal lavage. Postoperative adjuvant therapy was suggested but the patient refused. Now she showed no signs of recurrence 11 months after initial diagnosis, CA125and HE4 were both normal.

## Discussion and conclusions

Adenomyosis, a form of endometriosis, is a benign disorder in which the endometrium invades the myometrium and has a tendency towards malignant transformation [8]. Although adenomyosis is one of the most common benign histological manifestations in hysterectomy specimens, only 1% of cases undergo malignant transformation and form secondary endometrial carcinoma [1]. Since 1897, when the first case of EC-AIA was reported, no more than 100 cases have been noted. In an exploratory analysis, the author found that endometrioid and clear cell were the most common histologic types in EC-AIA patients [4].

At present, the pathological diagnostic criteria of EC-AIA are as follows [6]: (1) no tumour in the endometrium and other areas of the pelvic cavity, (2) tumour originating from adenomyotic epithelial cells with no invasion of other sites, and (3) abnormal glands around stromal cells of the endometrium. EC-AIA initially occurs only in the myometrium without endometrial involvement. Then, as the tumour progresses, the lesion may invade the endometrium. However, when the endometrium is involved, it is difficult to distinguish a cancerous invasion of adenomyosis from the situation in which endometrioid adenocarcinoma arising from the normally situated endometrium extends into foci of adenomyosis. In the present case, the normally situated endometrium was involved in the carcinoma, but the lesion in the myometrium was the main locus. Moreover, normal endometrial glands, atypical lesions, and cancerous cells can be seen in adenomyosis, which confirmed the diagnosis of EC-AIA.

Due to the rarity of this type of malignancy, there is a lack of standardized treatment for EC-AIA and the prognosis is not well characterized. For patients who are diagnosed with EC following hysterectomy, recommendations for further postoperative therapy are based on imaging (MRI and/or PET-CT) and on known risk factors for extrauterine disease related to the histologic grade and depth of myometrial invasion [9]. Individuals with high-risk factors (grade 3 lesions, deep myometrial invasion, or LVSI) may be candidates for additional surgery to remove the adnexa [9]. The national guideline recommends that lymphadenectomy is unnecessary for low-risk patients according to the Mayo triage algorithm but should be performed on individuals with high-risk factors [10]. According to the guideline [9], for patients with high/ intermediate risk factors (at least two of the factors: age > 60 years, deep myometrial invasion, grade 3, serous or clear cell histology, LVSI), vaginal brachytherapy provides excellent vaginal control. In this case, the patient was 69 years old and the myoinvasion was found deep in the myometrium(≥ 50% myometrium). Moreover, EC-AIA was found to be more likely with a poor prognosis [1]. So we made a final diagnosis of stage IB(G2) and judged the patient to have high/intermediate risk factors. Then, we performed additional operations including bilateral adnexectomy and pelvic and para-aortic lymphadenectomy. Postoperative adjuvant radiation therapy was suggested but the patient refused. Matsuo et al. [4] found that EC-AIA was more likely to be associated with deep myometrial invasion, high-grade, advanced stage, and lymph node metastasis, and there was no significant difference in prognosis between EC-AIA with and EC-AIA without endometrial involvement. This could be related to the fact that malignant cells in these cases are already located in the myometrium and can easily spread to the lymphatic and vascular systems [1]. Regular follow-up was performed on the patient in our case using imaging and tumor markers to determine the prognosis.

Although Endometrial carcinoma patients often have symptoms of vaginal bleeding or vaginal drainage, the clinical manifestation of EC-AIA is atypical. As a result, EC-AIA was often diagnosed in the hysterectomy specimen. Adequate investigations may contribute to the discovery of EC-AIA preoperatively. According to recent reports, the presence of recurrent unexplained bleeding, the identification of abnormal cells on cytology or the do novo symptom of dull pelvic pain in women who have additional risk factors for the endometrial carcinoma, calls for vigilance [11]. TVS and serum CA125 detection can be used as first-line investigation for EC-AIA. TVS has been extensively evaluated as an alternative method for identifying women at risk of endometrial disease by measuring the thickness of the endometrium [12]. A cut-off value of 5 mm is indicative of carcinoma in postmenopausal women with a high negative predictive value [9]. A higher index of suspicion is needed in the presence of thickened endometrium, a myometrial lesion, particularly one that increases in size, and/or elevated CA125, and further MRI examination is recommended. More recent case reports indicate that the lesions of EC-AIA can resemble degenerated fibroids radiologically, including MRI [11]. PET-CT is more helpful to determine the benign and malignant lesions. Then, in highly suspected malignancies, hysteroscopy provides an important diagnostic tool. On the one hand, hysteroscopy can directly and accurately observe the situation of endometrium and obtain the specimen of suspicious lesions. One the other hand, hysteroscopy can find the endometrial lesions when EC-AIA involves the endometrium.

In summary, endometrioid adenocarcinoma arising from adenomyosis is a rare entity. The early diagnosis of EC-AIA is difficult, especially when the lesion is confined to the foci of adenomyosis, which leads to a poor prognosis [1]. For postmenopausal patients with atypical symptoms, further detailed investigations need to be conducted with the consideration of EC-AIA. The treatment strategy should combine the stage, histopathologic grades and high-risk factors, and the formal guidelines still need further research. It will be necessary to accumulate a larger number of cases and more experience.

## Operational definition

*FIGO staging*: A staging system for cancer of the corpus uteri based on histologic verification of grading and extent of the tumor [9].

*POP*: It is defined as the descent of one or more of the anterior and posterior vaginal walls, uterus (cervix), or apex of the vagina (vaginal vault or cuff scar after hysterectomy) [13].

*POP-Q system*: POP-Q is defined by quantitative measurements of points representing the anterior, apical, and posterior walls of the prolapsed vagina. Stages are based on the maximal extent of prolapse relative to the hymen, in one or more compartments [13].

## Data Availability

The datasets used during the current study available from the corresponding author on reasonable request.

## Abbreviations

POP: Pelvic organ prolapse
EC-AIA: Endometrioid adenocarcinoma arising from adenomyosis
EC-A: Endometrial carcinoma coexisting with adenomyosis
EC: Endometrial carcinoma
BMI: Body mass index
POP-Q: Pelvic organ prolapse quantitation
TVS: Transvaginal ultrasonography
CT: Computed tomography
MRI: Magnetic resonance imaging
FIGO: International federation of gynecology and obstetrics
PET-CT: Positron-emission computed tomography
CA125: Carbohydrate antigen 125
HE4: Human epididymis protein 4
ER: Oestrogen receptor
PR: Progesterone receptor
CK-7: Cytokeratin 7
P53: Not applicable
P16: Not applicable
PAX-8: Not applicable
Ki-67: Not applicable
CEA: Carcinoembryonic antigen
LVSI: Lymphovascular space invasion
